# Beat-to-Beat Tracking of Pulse Pressure and Its Respiratory Variation Using Heart Sound Signal in Patients Undergoing Liver Transplantation

**DOI:** 10.3390/jcm8050593

**Published:** 2019-04-30

**Authors:** Yong-Seok Park, Young-Jin Moon, Sung-Hoon Kim, Jae-Man Kim, Jun-Gol Song, Gyu-Sam Hwang

**Affiliations:** Department of Anesthesiology and Pain Medicine, Biosignal Analysis and Perioperative Outcome Research Laboratory, University of Ulsan College of Medicine, Asan Medical Center, Seoul 05505, Korea; parkys@amc.seoul.kr (Y.-S.P.); yjmoon@amc.seoul.kr (Y.-J.M.); jaemankims@gmail.com (J.-M.K.); jungol.song@amc.seoul.kr (J.-G.S.); kshwang@amc.seoul.kr (G.-S.H.)

**Keywords:** heart sound, pulse pressure, liver transplantation, non-invasive monitor

## Abstract

Purpose: To investigate the possibility of esophageal phonocardiography as a monitor for invasively measured pulse pressure (PP) and its respiratory variation (PPV) in patients undergoing liver transplantation. Methods: In 24 liver transplantation recipients, all hemodynamic parameters, including PP and PPV, were measured during five predetermined surgical phases. Simultaneously, signals of esophageal heart sounds (S1, S2) were identified, and S1–S2 interval (phonocardiographic systolic time, PST) and its respiratory variation (PSV) within a 20-s window were calculated. Beat-to-beat correlation between PP and its corresponding PST was assessed during each time window, according to the surgical phases. To compare PPV and PSV along with 5 phases (a total of 120 data pairs), Pearson correlation was conducted. Results: Beat-to-beat PST values were closely correlated with their corresponding 3360 pairs of PP values (median *r* = 0.568 [IQR 0.246–0.803]). Compared with the initial phase of surgery, correlation coefficients were significantly lower during the reperfusion period (median *r* = 0.717 [IQR 0.532–0.886] vs. median *r* = 0.346 [IQR 0.037–0.677]; *p* = 0.002). The correlation between PSV and PPV showed similar variation according to the surgical phases (*r* = 0.576 to 0.689, *p* < 0.05, for pre-reperfusion; 0.290 to 0.429 for the post-reperfusion period). Conclusions: Continuous monitoring of intraoperative PST with an esophageal stethoscope has the potential to act as an indirect estimator of beat-to-beat arterial PP. Moreover, PSV appears to exhibit a trend similar to that of PPV with moderate accuracy. However, variation according to the surgical phase limits the merit of the current results, thereby necessitating cautious interpretation.

## 1. Introduction

Heart sounds result from the interplay of the dynamic events associated with the contraction and relaxation of the atria and ventricles, valve movement, and blood flow. A graphic recording of heart sounds, phonocardiography, provides valuable information concerning the function and integrity of the heart valves and on the hemodynamics of the heart, and has a high potential for detecting various heart diseases [1,2,3]. Although the esophageal stethoscope has been used for 60 years for continuous auscultation during general anesthesia [4], studies investigating phonocardiographic data as an indicator of cardiovascular function or continuous hemodynamic index are still very limited [5,6,7].

In our previous study, we suggested that intra-operative monitoring of the phonocardiogram may provide useful clinical information as a non-invasive hemodynamic index [8]. In that study, the time-domain analysis of heart sound signal was performed on digitalized recordings of in-house built esophageal stethoscopes in patients receiving general anesthesia. In specific, we had proposed phonocardiographically measured ventricular systolic time can be used as fluid responsiveness index in patient undergoing liver transplantation. In the current study, we further analyzed data to compare phonocardiographic signal derivatives with the corresponding beat-to-beat hemodynamic data including pulse pressure (PP) and its respiratory variation (PPV). As PP is proportional to stroke volume (SV), PP is clinically useful parameter to estimate a patient’s hemodynamic status. Furthermore, PPV is the one of the most popular hemodynamic index to indicate fluid responsiveness, and its clinical usefulness is without doubt. However, real-time arterial blood pressure monitoring through arterial cannulation is always necessary to obtain these parameters, making it difficult to apply to all cases. We believe non-invasive PPV monitoring helps patient care and eventually will improve patient outcome. Thus, the aim of this analysis was to assess whether intraoperative heart sound monitoring could be used as a surrogate for PP or PPV in patients undergoing major surgery.

## 2. Materials and Methods

### 2.1. Study Population and Anesthesia Protocol

This study was approved by the Institutional Review Board of Asan Medical Center (No. 2015-1371). The requirement for written informed consent was waived because of minimal risk to participants in this study. We reviewed electronic health records and routinely collected biosignal data of patients who underwent liver transplantation from June to September 2015. Patients with preoperative arrhythmia, valvular heart disease, reduced ventricular function (ejection fraction <40%), intracardiac shunt, hepatopulmonary syndrome, or pulmonary hypertension were excluded. Patients with incomplete signal data records were also excluded. The preoperative Child-Turcotte-Pugh class and Model for End-Stage Liver Disease (MELD) score were recorded.

All the patients were subjected to our institutional standard anesthetic management protocol for living donor liver transplantation [9,10,11,12]. Briefly, anesthesia was induced with thiopental, midazolam, fentanyl, and rocuronium, and was maintained using 1–1.5 vol% sevoflurane, 50% oxygen/air, and continuous infusion of fentanyl and rocuronium. Mechanical ventilation was performed without positive end-expiratory pressure, using a constant tidal volume of 8–10 mL/kg and a constant end-tidal carbon dioxide tension of 30–35 mmHg.

### 2.2. Hemodynamic Monitoring

After electrocardiography was applied, invasive radial arterial pressure was measured. The FloTrac/Vigileo device (software version 3.0, Edwards Lifescience, Irvine, CA, USA) was used to analyze invasive arterial pressure waveform data over 20-s intervals, with a recalibration interval of 1 min. A 7.5-Fr pulmonary arterial catheter (Swan-Ganz CCOmbo CCO/SvO2/CEDV, Edwards Lifescience), which was inserted via a 9-Fr introducer sheath into the internal jugular vein, was advanced to a wedged position under the guidance of a pressure curve recording. The pulmonary artery catheter was connected to a Vigilance device (Vigilance II, Edwards Lifescience). The Vigileo device computed stroke volume variation (SVV) from its relationship to the difference between the maximal and minimal values of stroke volume over a 20-s interval. We used a Multi-Data Logger (version 4.0, Edwards Lifescience) to capture and store patient data simultaneously from the Vigilance II and Vigileo devices. An 18 Fr esophageal stethoscope (DeRoyal Industries Inc., Powell, TN, USA) was inserted to monitor heart sound continuously during the operation. The esophageal stethoscope was positioned at the depth of 28–32 cm from upper incisor where the S1 sound was heard loudest, the location reported to be the best position to listen to heart sounds during the operation [13]. The depth of the ESS was then adjusted so that both the S1 and S2 heart sounds were clearly visible on the phonocardiogram. For phonocardiogram signal acquisition, a wireless digital stethoscope VPM2005W (Sunmeditec, Jeonju, Korea) was connected, and the beat-to-beat signal was recorded continuously at a 1000-Hz sampling rate. Digital signal analysis was conducted using CALC package of Advanced CODAS analysis software (version 3.25, Windaq, DATAQ Instruments, Akron, OH, USA), DADiSP (DSP Development, Cambridge, MA, USA), and Matlab R2010a (The MathWorks, Natick, MA, USA). 

### 2.3. Data Acquisition and Signal Processing

**Figure 1:**
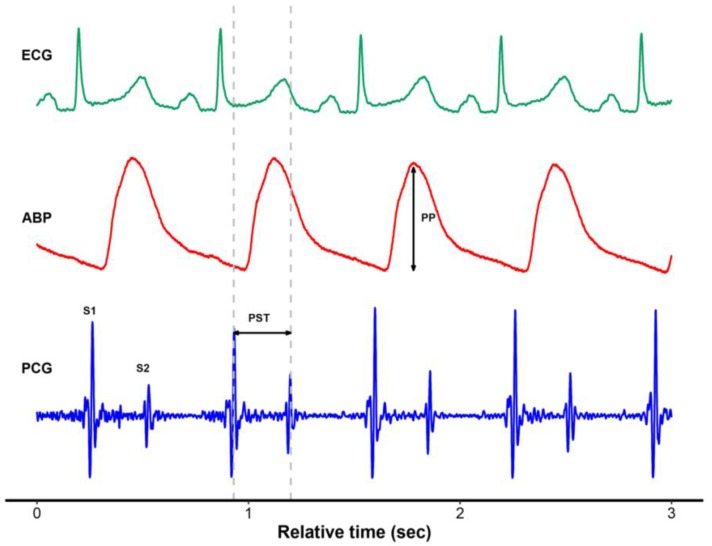
Representative plot of electrocardiogram, arterial blood pressure, and simultaneously recorded phonocardiogram using a digital esophageal stethoscope. ECG, electrocardiogram; ABP, arterial blood pressure; PCG, phonocardiogram; PP, pulse pressure; PST, phonocardiographic systolic time.

Hemodynamic parameters including blood pressure, heart rate (HR), central venous pressure (CVP), femoral venous pressure (FVP), pulmonary artery pressure, SV, cardiac output (CO), right ventricular end-diastolic volume index (RVEDVI), systemic vascular resistance (SVR) and phonocardiogram (PCG) signal were obtained. SV and CO were collected from STAT mode screen of the Vigilance monitor, which displayed them every 30–60 s. Based on our previous study [12], all data were recorded at the predetermined time points: 60 min after skin incision (I + 60), 30 min before clamping of inferior vena cava (IVC) (C – 30), 30 min after clamping of IVC (C + 30), 30 min after liver graft reperfusion (R + 30), and at the completion of hepatic artery reconstruction (HA). Phonocardiographic systolic time (PST) was defined as the interval between the first (S1) and second (S2) heart sounds (Figure 1). Pulse pressure (PP) was defined as the difference between systolic and diastolic pressure within a single curve. Beat-to-beat values of PST and PP were obtained at each time point for 20 s. Pulse pressure variation (PPV) was then calculated using following formula: PPV = (PPmax – PPmin) / [(PPmax + PPmin) / 2], where PPmax and PPmin were the maximal and minimal PP within a respiratory cycle [14]. Phonocardiographic systolic time variation (PSV) was calculated with the same process: PSV = (PSTmax – PSTmin) / [(PSTmax + PSTmin) / 2], where PSTmax and PSTmin were the maximal and minimal PST within a respiratory cycle. The values of the hemodynamic measurements obtained by averages taken over three respiratory cycles were used for statistical analysis. All data acquisition was performed by the same investigator.

When acquiring intraoperative PCG signal, a custom-built replica of a phonocardiography amplifier (VPM2005W, Sunmeditec, Jeonju, Korea) was used. The collection of data was conducted in a Windaq application (DATAQ Instruments, Akron, OH, USA) after which the data were stored on a database of the operating room. Raw PCG signal is not suitable for signal analysis because of various kinds of noises, including high frequency electrocautery signal and respiratory crackles during mechanical ventilation. Band-pass filtering (8–100 Hz) was applied using Matlab programming to remove white noise, and Hilbert transformation of the signal was performed to detect actual points of maximal amplitude of S1 and S2 heart sounds for appropriate digital signal analysis [8].

### 2.4. Statistical Analysis

Categorical variables were expressed as numbers and percentages. The hemodynamic data were expressed as mean ± standard deviation or median (IQR) as appropriate. The length of signal to be compared was selected to be at least three respiratory cycles. The beat-to-beat correlation between PP and its corresponding PST during each time window was evaluated in each patient using Pearson correlation coefficients. To compare between PSV and PPV for each phase, Pearson correlation and Bland–Altman analysis were conducted. A comparison between SVV and PSV was also performed. The *t*-test or rank sum test was used for comparison between the groups in each individual phase. All data analyses were performed using R version 3.5.0 (R Foundation for Statistical Computing, Vienna, Austria), and MedCalc version 13.1.1 (MedCalc Software, Mariakerke, Belgium). A *p* value < 0.05 was considered statistically significant.

## 3. Results

Electronic health records and anesthesia records of fifty-nine patients who underwent liver transplantation during the study period were reviewed. Among them, 18 cases were excluded due to patient factors (four with arrhythmia, two with valvular heart disease, two with reduced ventricular function, one with intracardiac shunt, one with hepatopulmonary syndrome, one with pulmonary hypertension, and seven cases in whom esophageal stethoscopes were not inserted because of the risk of bleeding from esophageal varices). Of the remaining, 17 cases were not eligible due to incomplete signal data (9 cases with poor quality of signal and 8 cases in which the predetermined time points were not marked).

As a result, the data of twenty-four recipients were analyzed. The demographic data and the preoperative evaluations of the patients in the study are presented in Table 1. A total of 3360 sets of beat-to-beat data points were identified with a median of 29 (IQR 27–31) data sets obtained from a 20 second window of each patient. During the overall operative period, most of hemodynamic variables at each phase were not significantly changed from the baseline values, except that the FVP was significantly higher and the RVEDVI was lower at C + 30 compared with the values at baseline time point (FVP, 25.8 ± 6.0 mmHg vs. 15.5 ± 4.2 mmHg, *p* < 0.001; RVEDVI, 121.5 ± 26.2 mL/m^2^ vs. 138.1 ± 27.5 mL/m^2^, *p* = 0.038). Additionally, PP was significantly higher at R + 30 compared with the baseline value (64.4 ± 16.2 mmHg vs. 54.6 ± 10.5 mmHg, *p* = 0.017) and PPV was significantly lower at R + 30 than the baseline (7.4 ± 4.7% vs. 5.1 ± 3.3%, *p* = 0.019). Table 2 shows hemodynamic data obtained at predetermined time points of five surgical phases of liver transplantation in 24 recipients.

**Figure 2:**
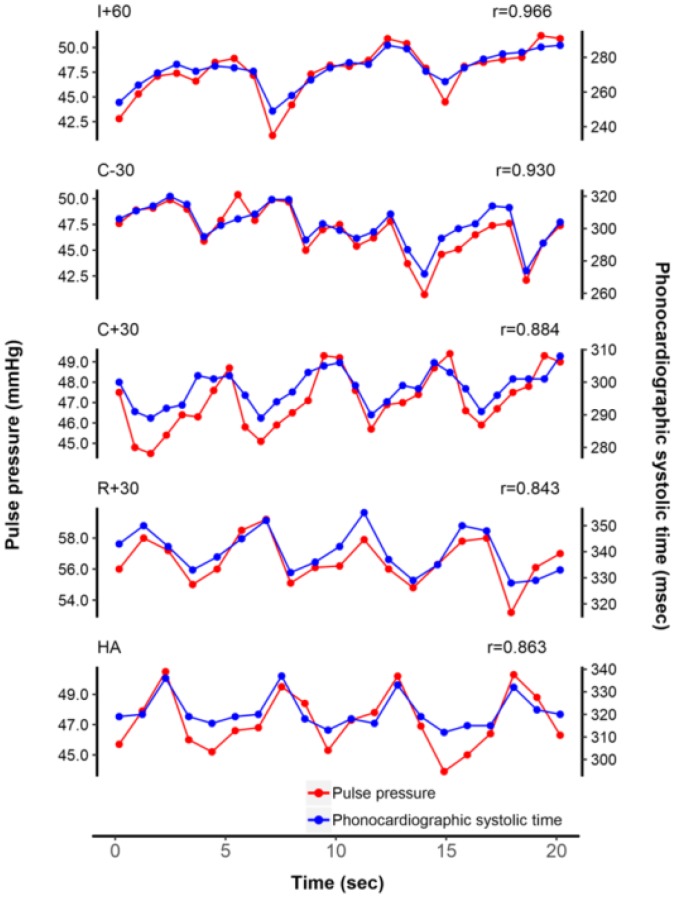
Representative tracing of beat-to-beat tracking of pulse pressure and phonocardiographic systolic time from an arbitrary patient. Note that the correlation coefficient decreased after reperfusion.

Representative plots of beat-to-beat tracking of PP and PST according to five surgical phases from a patient are shown on Figure 2. Beat-to-beat PST values were closely correlated with their corresponding PP values (all 3360 pairs of data, median *r* = 0.568 [IQR 0.246–0.803]). Compared with the correlation coefficients at I + 60, the correlation coefficients at R + 30 were significantly lower (I + 60, median *r* = 0.717 [IQR 0.532–0.886]; R + 30, median *r* = 0.346 [IQR 0.037–0.677]; *p* = 0.002) (Table 3). In all patients, median values of Pearson correlation coefficient between PST and PP at each phase were 0.717, 0.503, 0.611, 0.346, and 0.385, respectively. Correlation coefficients between beat-to-beat PST and PP from each of the 24 patients at five surgical phases are presented in Table 3.

**Figure 3:**
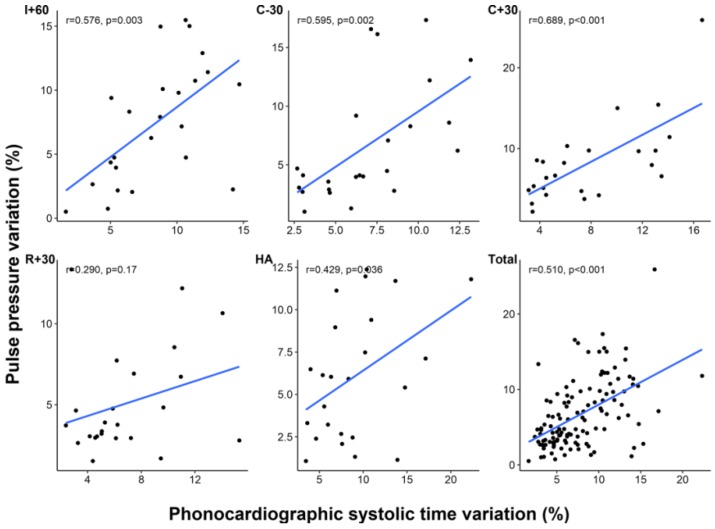
Correlation between phonocardiographic systolic time variation and pulse pressure variation at each time point. I + 60, 60 min after skin incision; C − 30, 30 min before clamping of inferior vena cava; C + 30, 30 min after clamping of inferior vena cava; R + 30, 30 min after liver graft reperfusion; HA, at the completion of hepatic artery reconstruction.

**Figure 4:**
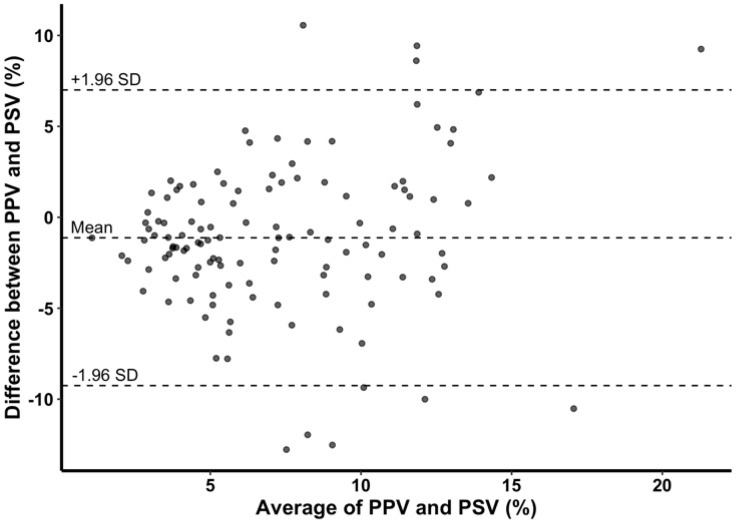
Bland–Altman plot of difference between pulse pressure variation (PPV) and phonocardiographic systolic time variation (PSV) and average of PPV and PSV. Mean difference between PPV and PSV was −1.1% with 95% limits of agreement of −9.3% to 7.0%.

PSV and PPV showed significant correlation at most of the time points except at R + 30 (all time points, *r* = 0.510, *p* < 0.001; R + 30, *r* = 0.290, *p* = 0.170) (Figure 3). In Bland–Altman analysis, mean difference between PPV and PSV was –1.1% with 95% limits of agreement of –9.3% to 7.0% (Figure 4). Although SVV and PSV also showed significant correlation at time points before graft reperfusion (all time points, *r* = 0.319, *p* < 0.001), the correlation was weaker than that between PPV and PSV, and there was no significant correlation between SVV and PSV at timepoints after reperfusion (R + 30, *r* = 0.335, *p* = 0.109; HA, *r* = 0.055, *p* = 0.797) (Table 4).

## 4. Discussion

In this study, we hypothesized that heart sound signal monitoring using an esophageal stethoscope would be a useful non-invasive hemodynamic index. Specifically, the S1–S2 interval, measured as the PST, was closely correlated with corresponding beat-to-beat arterial PP, and the correlation was more evident at the other timepoints than at the post-reperfusion period. Furthermore, respiratory variation of S1–S2 interval (PSV) has similar incremental/decremental pattern according to hemodynamic fluctuation compared with PPV as well. 

Although esophageal stethoscopes have been used for 60 years for continuous auscultation during general anesthesia [4,15,16,17], studies investigating phonocardiographic data as intraoperative hemodynamic parameters or continuous monitoring modalities are still limited. In our previous study, we suggested that intra-operative monitoring of phonocardiogram may provide useful information for non-invasive hemodynamic index [8]. Specifically, typical components in the phonocardiographic signal, including S1–S2 interval and its respiratory variation may have potential for fluid responsiveness index. In the current study, we assumed that more blood volume in the ventricle to be ejected (more SV) needs more ejection time, which is represented by PST. Because PP is known to be proportional to SV, we hypothesized that PST is proportional to PP. In accordance with this, the most interesting finding of the current study was that non-invasively measured PST can successfully track invasively measured arterial PP in beat-to-beat manners. Moreover, PSV, the respiratory variation of PST, was shown to be a possible non-invasive surrogate for PPV, which is a dynamic index for fluid responsiveness. PPV is the one of the most important monitoring parameters during surgical procedure to maintain optimal fluid administration. However, invasiveness of PPV monitoring limits the clinical application and is regarded as main drawback. Our results suggested that the parameters derived from non-invasive heart sound recordings have possibilities to be a good substitute for the hemodynamic parameters from invasive monitoring. We think PST or PSV can be used to monitor the changing trends in SV or to determine fluid responsiveness in a clinical situation that the invasive monitoring is not possible or not prepared.

Our results show that the performance in tracking the beat-to-beat PP varies depending on the surgical phases and individual characteristics. In most patients, PST and PP showed good correlation before graft reperfusion, but the correlations were significantly lower at the timepoints after reperfusion. In addition, there were two patients who showed unfavorable correlations in most timepoints, regardless of the surgical phase (patient #5 and #13 on Table 3). Patient #5 was a 64-year-old female who underwent liver transplantation due to primary sclerosing cholangitis with advanced liver cirrhosis (MELD score, 24; total bilirubin, 38.1 mg/dL). In this patient, PP was high (80.9, 83.9, 78.7, 94.5, and 100.3 mmHg at each timepoint) and diastolic blood pressure was low (46.8, 55.6, 56.4, 56.3, and 58.3 mmHg at each timepoint) throughout the surgery, suggesting SV increase and SVR decrease due to hemodynamic changes in the cirrhotic patient. In the same vein, SVR measured with the Vigilance device was also low (409, 533, 590, 598, and 512 dyne·s/cm^5^ at each timepoint) in this patient. It has been known that the PP depends not only on SV but also on properties of arterial circulation such as aortic compliance [18]. These factors could be altered in cirrhotic patients because they usually have high CO and low SVR [19] (low SVR means that there is an overall relative vasodilatation, which would explain the resultant lesser degree of rebound of the elastic tissue of the resistance arterioles hence resulting in a lower diastolic pressure). Theoretically, vascular tone might have been an influence on the accuracy of dynamic indices [20,21]. For example, Vigileo devices that measure cardiac output through analysis of arterial pulse wave had been tested during liver transplantation and shown to have a low accuracy when compared with thermodilution [22,23]. The authors hypothesized that the cardiomyopathy and vascular compliance change of cirrhotic patients was likely to be responsible for such findings, particularly in patients with high grade of Child–Pugh score and high MELD score [22,23]. Furthermore, this hypothesis is consistent with the fact that the PST–PP correlation has weakened after hemodynamic changes (including increase in PP, probably partly due to reduction of SVR) after reperfusion in most patients in our current study. In case of patient #13, however, the features consistent with this hypothesis are less clear (PP, 58.8, 50.3, 58.3, 49.2, and 48.8 mmHg; diastolic blood pressure, 71.1, 53.9, 68.9, 57.6, and 56.3 mmHg; SVR, 733, 440, 572, 543, and 599 dyne·s/cm^5^ at each time point). In this case, the individual characteristics of the patient, an error in measurement, or a limitation of the signal processing method may be considered as the cause.

There were several limitations to our study. First, the patient population of the present study was only limited to cirrhotic patients undergoing liver transplantation. As patients with end-stage liver disease usually show altered hemodynamic characteristics such as low SVR and underlying cirrhotic cardiomyopathy [24,25], the results of the current study may not be generalized to patients with normal cardiovascular function. Second, although we used Hilbert transformation to detect peak points of S and S2, this was not validated for phonocardiographic signal analysis. Furthermore, the band-pass filters significantly reduced noise, but such processing can induce signal distortion as well. Therefore, the different effects between signal filters should be cautiously evaluated in any further study. Third, the correlation between SVV and PSV was weaker than that between PPV and PSV, although SVV is known as indicator of fluid responsiveness. We acquired SVV values from the Vigileo device which estimates SV from arterial waveform by its own algorithm and then calculates SVV from the estimated SV values. It is possible that there was a difference between the estimated SV and real SV. Meanwhile, PPV was directly calculated from PP which is measured in beat-to-beat manner, without time delay. Direct measurement of SV using doppler device or echocardiography may help to reduce this gap between SVV and PPV. Lastly, this was a small, single-center study. Further large-scale studies are required to evaluate the full clinical impacts of PST and PSV in clinical anesthesia.

In conclusion, non-invasive determination of PST and PSV parameters using heart sounds can provide useful continuous hemodynamic index during liver transplantation. Intraoperative monitoring of PST and PSV will enable the clinicians in estimating the cardiovascular status of surgical patients and eventually reduce uncertainties in the bedside assessment of preload status. Further research and development of this technique is warranted.

## Figures and Tables

**Table 1:** Demographic data and preoperative findings of 24 liver transplant recipients.

Characteristics	
Sex (M/F)	19/5
Age (years)	55.0 ± 10.4
Weight (kg)	66.1 ± 13.3
Height (cm)	164.5 ± 8.0
Classification of liver transplant recipients	
Hepatitis virus-related liver cirrhosis	12 (50.0%)
Alcoholic cirrhosis	6 (25.0%)
Secondary biliary cirrhosis	2 (8.4%)
Autoimmune or cryptogenic cirrhosis	3 (12.5%)
Acute hepatitis	1 (4.2%)
Child-Turcotte-Pugh score	8.2 ± 1.5
Model for End-Stage Liver Disease score	15.2 ± 6.9
Preoperative transthoracic echocardiographic findings	
Ejection fraction (%)	65.2 ± 4.3
End-diastolic volume (mL)	116.1 ± 31.3
End-systolic volume (mL)	40.5 ± 12.3
Stroke volume (mL)	75.6 ± 21.1
Left ventricular mass (g)	162.9 ± 43.5
Preoperative hematologic profiles	
Hemoglobin (g/dL)	10.7 ± 1.6
Platelet count (×10^3^/mm^3^)	86.3 ± 68.5
Prothrombin time (international normalized ratio)	1.6 ± 0.6
Albumin (g/dL)	3.1 ± 0.5
Creatinine (mg/dL)	1.0 ± 1.3
Bilirubin (mg/dL)	5.4 ± 8.9

Values represent number of recipients or mean ± SD.

**Table 2:** Hemodynamic data obtained at five predetermined time points during liver transplantation in 24 recipients.

Parameter	I + 60	C − 30	C + 30	R + 30	HA
SBP (mmHg)	114.3 ± 14.5	114.1 ± 17.1	115.4 ± 13.5	123.5 ± 27.0	118.6 ± 16.2
DBP (mmHg)	59.6 ± 10.0	58.6 ± 9.8	63.3 ± 8.3	59.8 ± 14.2	57.3 ± 7.7
HR (beats/min)	91.7 ± 12.1	91.1 ± 14.6	94.7 ± 14.6	91.7 ± 14.0	89.1 ± 12.3
CVP (mmHg)	11.4 ± 2.5	10.7 ± 2.2	11.0 ± 2.6	12.3 ± 2.6	11.6 ± 1.9
FVP (mmHg)	15.5 ± 4.2	13.8 ± 2.4	25.8 ± 6.0 *	15.0 ± 3.5	14.7 ± 3.2
CO (L/min)	8.0 ± 2.3	8.3 ± 2.5	6.9 ± 2.3	9.0 ± 2.5	8.3 ± 2.5
SV (mL/beat)	87.9 ± 25.9	90.6 ± 27.8	78.1 ± 28.8	94.4 ± 26.0	94.7 ± 26.8
RVEDVI (mL/m^2^)	138.1 ± 27.5	132.4 ± 24.9	121.5 ± 26.2 *	131.9 ± 30.9	131.7 ± 29.4
SVR (dyne·s/cm^5^)	718.0 ± 236.2	711.5 ± 245.7	711.2 ± 298.6	679.9 ± 224.8	673.1 ± 251.6
PP (mmHg)	54.6 ± 10.5	55.8 ± 11.8	51.5 ± 11.4	64.4 ± 16.2 *	61.4 ± 1.37
SVV (%)	8.5 ± 3.9	8.4 ± 4.3	11.3 ± 7.6	6.6 ± 3.3	8.4 ± 2.4
PPV (%)	7.4 ± 4.7	6.7 ± 5.0	8.2 ± 5.1	5.1 ± 3.3 *	6.1 ± 3.8
PST (ms)	279.4 ± 40.8	275.1 ± 36.8	259.3 ± 42.7	286.8 ± 39.5	269.1 ± 40.7
PDT (ms)	387.7 ± 80.9	386.8 ± 73.6	375.2 ± 62.8	396.5 ± 92.6	426.9 ± 97.5
PSV (%)	8.4 ± 3.4	7.0 ± 3.2	7.8 ± 4.2	6.9 ± 3.5	9.1 ± 4.6
S1 amplitude (dB)	26.6 ± 4.9	26.7 ± 5.4	25.3 ± 5.1	25.0 ± 6.1	25.1 ± 6.9
S2 amplitude (dB)	22.4 ± 4.6	21.8 ± 4.7	20.0 ± 4.9 *	20.7 ± 5.2	21.0 ± 5.5

Data are presented as mean ± SD. SBP, systolic blood pressure; DBP, diastolic blood pressure; HR, heart rate; CVP, central venous pressure; FVP, femoral venous pressure; CO, cardiac output; SV, stroke volume; RVEDVI, right ventricular end-diastolic volume index; SVR, systemic vascular resistance; PP, pulse pressure; SVV, stroke volume variation; PST, phonocardiographic systolic time; PDT, phonocardiographic diastolic time; PSV, phonocardiographic systolic time variation; S1amp, I + 60, 60 min after skin incision; C – 30, 30 min before clamping of inferior vena cava; C + 30, 30 min after clamping of inferior vena cava; R + 30, 30 min after liver graft reperfusion; HA, at the completion of hepatic artery reconstruction. * *p* < 0.05 compared with hemodynamic values obtained at the I + 60 time point.

**Table 3:** Correlations between beat-to-beat pulse pressures and their corresponding phonocardiographic systolic times obtained at five predetermined time points during liver transplantation in 24 recipients.

Patient	I + 60	C − 30	C + 30	R + 30	HA
1	0.469 **	0.877 ***	0.622 ***	0.689 ***	0.633 ***
2	0.761 ***	0.435 *	0.904 ***	0.043	0.619 **
3	0.791 ***	0.956 ***	0.817 ***	0.903 ***	0.831 ***
4	0.611 ***	0.326	0.543 **	−0.067	0.820 ***
5	−0.155	−0.155	0.722 ***	−0.324	−0.361
6	0.643 ***	−0.186	0.429 *	0.318	0.709 ***
7	0.896 ***	0.884 ***	0.799 ***	0.497 **	0.376
8	0.748 ***	0.819 ***	0.859 ***	−0.229	0.891 ***
9	0.535 **	0.031	0.839 ***	−0.018	0.067
10	0.931 ***	0.552 **	0.172	0.022	−0.412 *
11	0.621 ***	0.354	0.076	0.769 ***	0.394 *
12	0.650 ***	0.518 **	0.252	−0.001	0.583 **
13	0.187	−0.035	0.638 ***	0.110	0.078
14	0.687 ***	0.889 ***	0.583 **	0.305	0.333
15	0.950 ***	0.944 ***	0.768 ***	0.191	0.774 ***
16	0.435 *	0.371	0.411	0.664 **	0.226
17	0.907 ***	0.034	0.885 ***	0.374	0.062
18	0.965 ***	0.948 ***	0.883 ***	0.844 ***	0.863 ***
19	0.758 ***	0.488 *	0.479*	0.673 ***	−0.298
20	0.772 ***	0.248	0.600 **	0.834 ***	0.745 ***
21	0.478 **	0.253	−0.401	0.389 *	0.376 *
22	0.921 ***	0.942 ***	0.238	0.754 ***	0.927 ***
23	0.882 ***	0.821 ***	−0.189	0.066	0.355 *
24	0.524 **	0.620 ***	0.643 ***	0.515 **	0.125
Median, IQR	0.717 (0.532–0.886)	0.503 (0.252–0.879)	0.611 (0.372–0.803)	0.346 (0.037–0.677)	0.385 (0.113–0.753)

All values are shown as Pearson correlation coefficients; I + 60, 60 min after skin incision; C − 30, 30 min before clamping of inferior vena cava; C + 30, 30 min after clamping of inferior vena cava; R + 30, 30 min after liver graft reperfusion; HA, at the completion of hepatic artery reconstruction. * *p* < 0.05; ** *p* < 0.01; *** *p* < 0.001.

**Table 4:** Correlation coefficients between phonocardiographic systolic time variation, pulse pressure variation, and stroke volume variation obtained at five predetermined time points during liver transplantation in 24 recipients.

		I + 60	C − 30	C + 30	R + 30	HA
PPV vs. PSV	*r*	0.576	0.595	0.689	0.290	0.429
	*p*	0.003	0.002	<0.001	0.170	0.036
SVV vs. PSV	*r*	0.407	0.408	0.407	0.335	0.055
	*p*	0.048	0.048	0.048	0.109	0.797

PPV, pulse pressure variation; PSV, phonocardiographic systolic time variation; SVV, stroke volume variation; I + 60, 60 min after skin incision; C − 30, 30 min before clamping of inferior vena cava; C + 30, 30 min after clamping of inferior vena cava; R + 30, 30 min after liver graft reperfusion; HA, at the completion of hepatic artery reconstruction.
